# Genomics Assisted Ancestry Deconvolution in Grape

**DOI:** 10.1371/journal.pone.0080791

**Published:** 2013-11-11

**Authors:** Jason Sawler, Bruce Reisch, Mallikarjuna K. Aradhya, Bernard Prins, Gan-Yuan Zhong, Heidi Schwaninger, Charles Simon, Edward Buckler, Sean Myles

**Affiliations:** 1 Department of Plant and Animals Sciences, Faculty of Agriculture, Dalhousie University, Truro, Nova Scotia, Canada; 2 Department of Horticulture, Cornell University, Ithaca, New York, United States of America; 3 National Clonal Germplasm Repository, United States Department of Agriculture, Agricultural Research Service, University of California, Davis, Davis, California, United States of America; 4 United States Department of Agriculture, Agricultural Research Service, Grape Genetics Research Unit, New York State Agricultural Experiment Station, Cornell University, Geneva, New York, United States of America; 5 United States Department of Agriculture, Agricultural Research Service, Plant Genetic Resources Unit, New York State Agricultural Experiment Station, Cornell University, Geneva, New York, United States of America; 6 Department of Plant Breeding and Genetics, Cornell University, Ithaca, New York, United States of America; 7 United States Department of Agriculture, Agricultural Research Service, Robert W. Holley Center for Agriculture and Health, Ithaca, New York, United States of America; Agriculture and Agri-Food Canada, Canada

## Abstract

The genus *Vitis* (the grapevine) is a group of highly diverse, diploid woody perennial vines consisting of approximately 60 species from across the northern hemisphere. It is the world’s most valuable horticultural crop with ~8 million hectares planted, most of which is processed into wine. To gain insights into the use of wild *Vitis* species during the past century of interspecific grape breeding and to provide a foundation for marker-assisted breeding programmes, we present a principal components analysis (PCA) based ancestry estimation method to calculate admixture proportions of hybrid grapes in the United States Department of Agriculture grape germplasm collection using genome-wide polymorphism data. We find that grape breeders have backcrossed to both the domesticated *V. vinifera* and wild *Vitis* species and that reasonably accurate genome-wide ancestry estimation can be performed on interspecific *Vitis* hybrids using a panel of fewer than 50 ancestry informative markers (AIMs). We compare measures of ancestry informativeness used in selecting SNP panels for two-way admixture estimation, and verify the accuracy of our method on simulated populations of admixed offspring. Our method of ancestry deconvolution provides a first step towards selection at the seed or seedling stage for desirable admixture profiles, which will facilitate marker-assisted breeding that aims to introgress traits from wild *Vitis* species while retaining the desirable characteristics of elite *V. vinifera* cultivars.

## Introduction

 The genus *Vitis* (the grapevine) is a group of highly diverse, diploid woody perennial vines consisting of approximately 60 species from across the northern hemisphere [1]. According to the archaeological record, cultivation of the domesticated grapevine, *Vitis vinifera*, began 6000-8000 years ago in the Near East [2]. Today, the grape is the world’s most valuable horticultural crop with ~8 million hectares planted, most of which is processed into wine (http://faostat.fao.org; Accessed 2013 October 21). Grapes from the domesticated species, *V. vinifera*, account for more than 95% of the grapes grown worldwide [1] and the world’s vineyards are dominated by a small number of closely related *V. vinifera* cultivars that have often been vegetatively propagated for centuries [3]. Because they are perpetually propagated, elite grape cultivars require increasingly intense chemical applications to combat evolving pathogen pressures. It is widely recognized that the exploitation of wild *Vitis* species’ resistance to disease is crucial to the continued success and expansion of the grape and wine industries, and that the grape is well-poised to benefit from the use of marker-assisted breeding for this purpose [1,4,5]. 

 In plant breeding, marker-assisted backcrossing can be used to incorporate traits into elite cultivars while minimizing the transfer of undesirable alleles from the donor genome [6]. This process involves both foreground and background selection. Foreground selection refers to the screening and selection of offspring based on the presence or absence of a specific allele that is associated with a trait of interest. In contrast, background selection is the selection of offspring on the basis of genomic ancestry estimates. A breeder may wish to introgress a specific trait from a wild species into an elite cultivar, while minimizing the genomic contribution from the wild species unrelated to that trait [4,5]. Recombinant selection (through backcrossing) aims to reduce the size of the chromosomal segment carrying the desired locus. Wild species often possess genes that negatively affect crop performance, making it advantageous to remove any additional background contribution from these wild species to the genomes of the resulting progeny [6]. While backcrossing in many crops is performed by crossing offspring back to one of the parents, “pseudo-backcrossing” is the method used to perform backcrosses in grapes. Pseudo-backcrossing involves crossing hybrid offspring back to a cultivated *V. vinifera* cultivar that is not one of the parents from the original cross. This form of backcrossing is performed because grapes suffer from severe inbreeding depression and thus crosses between closely related cultivars must be avoided [5]. Background selection relies on accurate estimation of the percentages of the donor and recurrent parental genomes present in the resulting progeny.

 To gain insights into the use of wild *Vitis* species during the past century of interspecific grape breeding and to provide a foundation for background selection in marker-assisted breeding programmes, we present a principal components analysis (PCA) based ancestry estimation method to calculate admixture proportions of hybrid grapes in the US Department of Agriculture (USDA) grape germplasm collection using genome-wide polymorphism data from the Vitis9kSNP microarray [3]. We find that grape breeders have backcrossed to both *V. vinifera* and wild *Vitis* species and that reasonably accurate genome-wide ancestry estimation can be achieved in interspecific *Vitis* hybrids using a panel of fewer than 50 ancestry informative markers (AIMs). Our method of ancestry deconvolution provides a first step towards selection at the seed or seedling stage for desirable admixture profiles, which will facilitate marker-assisted breeding that aims to introgress traits from wild *Vitis* species while retaining the desirable characteristics of elite *V. vinifera* cultivars.

## Materials and Methods

### Sample Collection and Genotype Calling

Leaf tissue was collected from the USDA grape germplasm collections in Davis, California and Geneva, New York. Permission for tissue collection was obtained from the local USDA authority. DNA was extracted using commercial extraction kits. Genotype data were generated from the custom Illumina Vitis9KSNP array, which assays 8,898 single nucleotide polymorphisms (SNPs). After quality filters (GenTrain Score ≥ 0.3 and GenCall ≥ 0.2) 6114 SNPs in 1817 *Vitis* samples remained for analysis [3].

### Data curation

Samples with >10% missing data were removed, and SNPs with >10% missing data and minor allele frequency (MAF) <0.10 were removed using PLINK [7]. After these filters, 1599 samples and 2959 SNPs remained. PCA was performed on this data set using SMARTPCA [8] and 60 samples were removed due to mislabeling. For example, some samples labeled as *V. vinifera* clustered with wild species and some samples labeled as hybrids clustered with wild or *V. vinifera* (Figure S1). DNA sample mix-up is an unlikely explanation for these errors because sample processing was done primarily with robotics and no genotype discordance for 145 pairwise comparisons between replicate samples placed randomly across sample plates was observed [3]. Thus, the cases of mislabeling are likely due to curation error. Our ancestry estimates of putatively mislabeled individuals are currently being verified by direct observation in the vineyard and the USDA Germplasm Resources Information Network (GRIN) online database [9] will be updated accordingly. The data are available from the Dryad Digital Repository: http://dx.doi.org/10.5061/dryad.45hh0.

Our PCA plot of the full data set revealed a clear separation of North American wild species from *V. vinifera* along the first principal component (PC1; Figure S1). Eurasian wild species fell between these two groups. Although they are occasionally used in grape breeding, we excluded Eurasian wild species and hybrids with known Eurasian wild ancestry from the remaining analysis because the number of samples was low and their position in PC space complicates ancestry estimation. The present study thus focuses on hybrids with ancestry from North American wild *Vitis* species (hereafter referred to simply as wild *Vitis*) and *V. vinifera*.

### Admixture analyses

**Figure 1:**
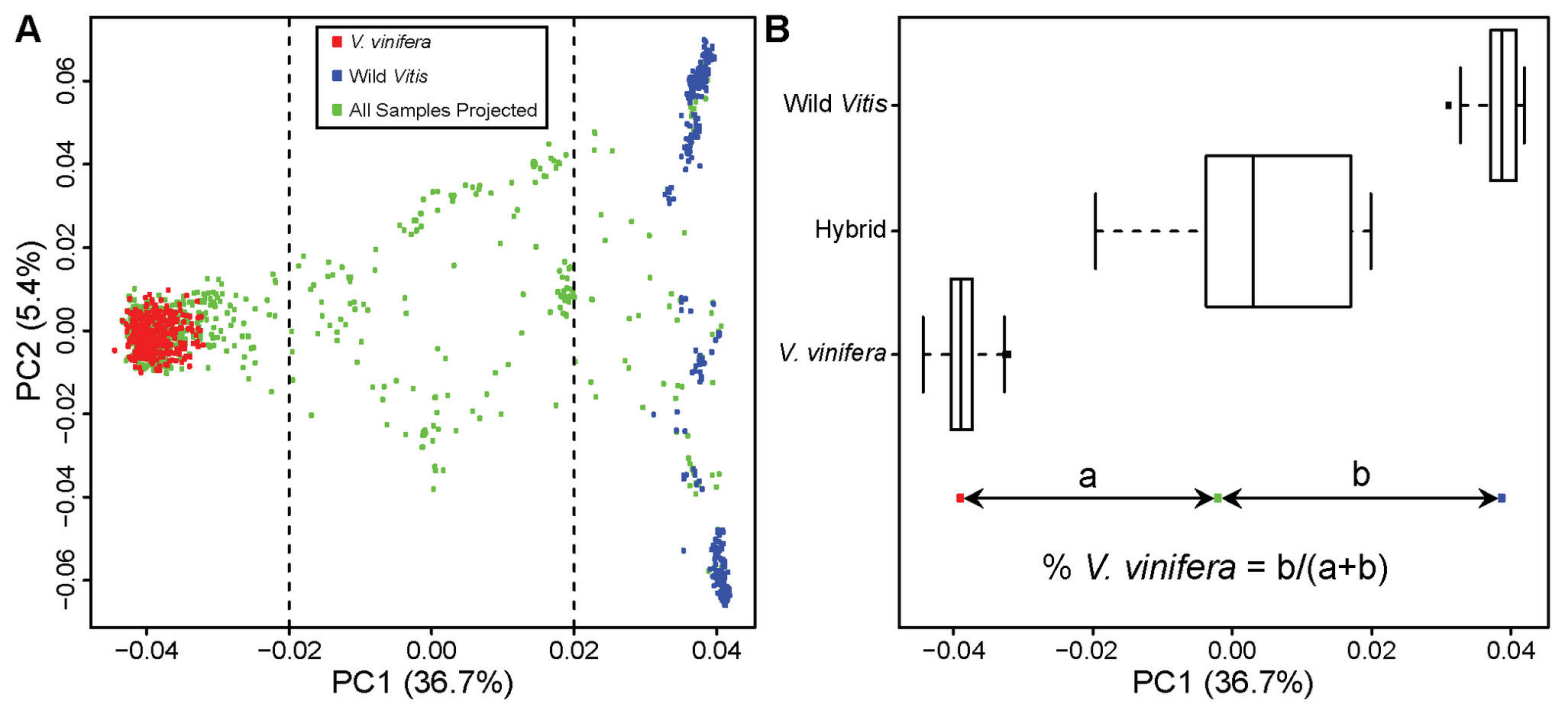
PCA based ancestry estimation. (A) PC axis 1 (PC1) and PC2 were calculated using 2959 SNPs from 333 *V. vinifera* and 333 wild *Vitis* samples. The proportion of the variance explained by each PC is shown in parentheses along each axis. Subsequently, 1599 samples, including various *Vitis* species and hybrids, were projected onto these axes (green dots). Samples lying between the dotted vertical lines were considered hybrids for the remaining analyses. (B) Boxplots show the range of PC1 values for the two ancestral populations (*V. vinifera* and wild *Vitis*) and the hybrids identified in (A). Boxes denote upper and lower quartiles and whiskers extend to 2.7 SD. Below the boxplots, an illustration of how ancestry proportions are calculated is provided (see Methods for details).

Principal components were computed using 333 wild *Vitis* samples and a random sample of 333 *V. vinifera* samples. Equal sample sizes (N = 333) for ancestral populations were selected as this has been shown to be a crucial factor in accurately inferring genetic relatedness based on PCA [10]. After establishing the PC axes based on these ancestral populations, all 1599 samples were subsequently projected on to these axes, and individuals between -0.02 and 0.02 on PC1 (with the exception of Eurasian wild samples and hybrids with known Eurasian wild ancestry) were considered hybrids for the remainder of the analysis (N = 127 hybrids; Figure 1A). These conservative thresholds were chosen because the projection space between them included all samples labeled as “hybrid” in the USDA Germplasm Resources Information Network database [9].

Our method of calculating ancestry coefficients employs the approach described in [11,12], where admixture proportions are equal to the coordinate distance in PC space between the admixed individual (hybrid) and the two ancestral populations (*V. vinifera* and wild *Vitis*). For each purported hybrid grape, the genome-wide proportion of *V. vinifera* is estimated as P = b/(a+b), where b and a are the chord distances from the wild *Vitis* and *V. vinifera* centroids, respectively, for the given hybrid along PC1 (Figure 1B). We also estimated ancestry proportions using the model-based software STRUCTURE [13]. STRUCTURE was run with a burn-in period of 20,000 iterations followed by 100,000 iterations using the admixture model where each sample draws some fraction of its genome from each of the K populations where K = 2. As with previous work [14], our PCA-based ancestry estimates are highly similar to those generated from STRUCTURE (R^2^ = 0.998; Figure S2). 

### Measures of Ancestry Informativeness

 We ranked 2959 SNPs according to four measures of ancestry informativeness: PC1 Weight, PC1 Positive Weight, F_ST_ and a linear model described below. We evaluated the ability of reduced marker sets (1-100 SNPs) based on these measures to predict ancestry relative to the full set of 2959 SNPs. The “classification accuracy” of a set of AIMs is the R^2^ value generated from a Pearson correlation between the hybrid ancestry estimates based on the reduced marker set and the estimates based on the full set of 2959 SNPs. PC1 Weight is the absolute value of the PC1 loading given to each SNP by SMARTPCA, whereas PC1 Positive Weight excludes any values given a negative loading by the software. F_ST_, a measure of allele frequency difference between the ancestral populations, was calculated according to [15] for each SNP using allele frequencies output by PLINK. For the Linear Model (LM) measure, linear regression was performed in R [16] using genome-wide *V. vinifera* content (estimated from all 2959 markers) as a response variable and genotypes for a given SNP across wild *Vitis*, *V. vinifera* and hybrid samples as an explanatory variable (0 = homozygous reference allele; 1 = heterozygous; 2 = homozygous non-reference allele). SNPs were ranked according to their R^2^ from the linear model as a measure of ancestry informativeness.

### Simulations of Admixture

 To evaluate the accuracy of our PCA-based ancestry estimation method, *in silico* crosses between *V. vinifera* and wild species were simulated in R. Simulated F1 offspring were generated by randomly sampling one of the 333 *V. vinifera* and one of the 333 wild *Vitis* as parents. Parental genotypes were combined to produce offspring genotypes by sampling one allele at random from each parent at each SNP. Linkage disequilibrium between SNPs was ignored. This procedure was repeated 10,000 times to generate 10,000 F1 offspring. To generate simulated F2 populations this process was repeated, using the F1 individuals as one ancestral population and either wild or *V.vinifera* accessions as the other to simulate backcrossing (n = 10000 for F1 backcrossed to wild, and n = 10000 for F1 backcrossed to *V. vinifera*).

## Results and Discussion

### PCA-based ancestry estimation

 PCA is a useful tool for revealing patterns of population structure and relatedness among samples for which genome-wide SNP data are available [17-19]. A genotyping microarray for the grape, the Vitis9KSNP array, was recently developed with probes designed for SNPs segregating within the domesticated species, *V. vinifera*, and a small number of probes designed to assay variation among *Vitis* species [20]. When PCA is applied to Vitis9KSNP array data from a diverse collection of *V. vinifera* cultivars, wild *Vitis* species and hybrid cultivars, PC1 clearly separates wild *Vitis* species from *V. vinifera* while hybrid cultivars lie between these two groups (Figure 1A, S1). This observation motivated us to apply methods developed previously [11,12] to use a hybrid cultivar’s projected position along PC1 to estimate the proportion of its ancestry derived from *V. vinifera* and wild *Vitis* species (Figure 1B). Our PCA-based method provides highly similar ancestry estimates to those generated from the model-based approach in STRUCTURE (Figure S2).

 For the present study, we considered a sample a “hybrid” if its projected position along PC1 was between -0.02 and 0.02. After removing obvious errors (see Methods), all samples labeled as “hybrid” in the USDA Germplasm Resources Information Network (GRIN) online database [9] fall within this range, but many samples labeled as either wild *Vitis* species or *V. vinifera* fall within this range as well (Figure S1). Our ancestry estimates are therefore being used to improve the accuracy of the ancestry assignments associated with each accession in the USDA grape germplasm collection. We acknowledge, however, that samples outside of our defined “hybrid” range may in fact represent hybrid samples that resulted from extensive backcrossing to either *V. vinifera* or wild *Vitis* species. Further studies will be required to verify the ancestry of such hybrid samples and distinguish them unequivocally from the ancestral groups.

### Verification of ancestry estimation method

**Figure 2:**
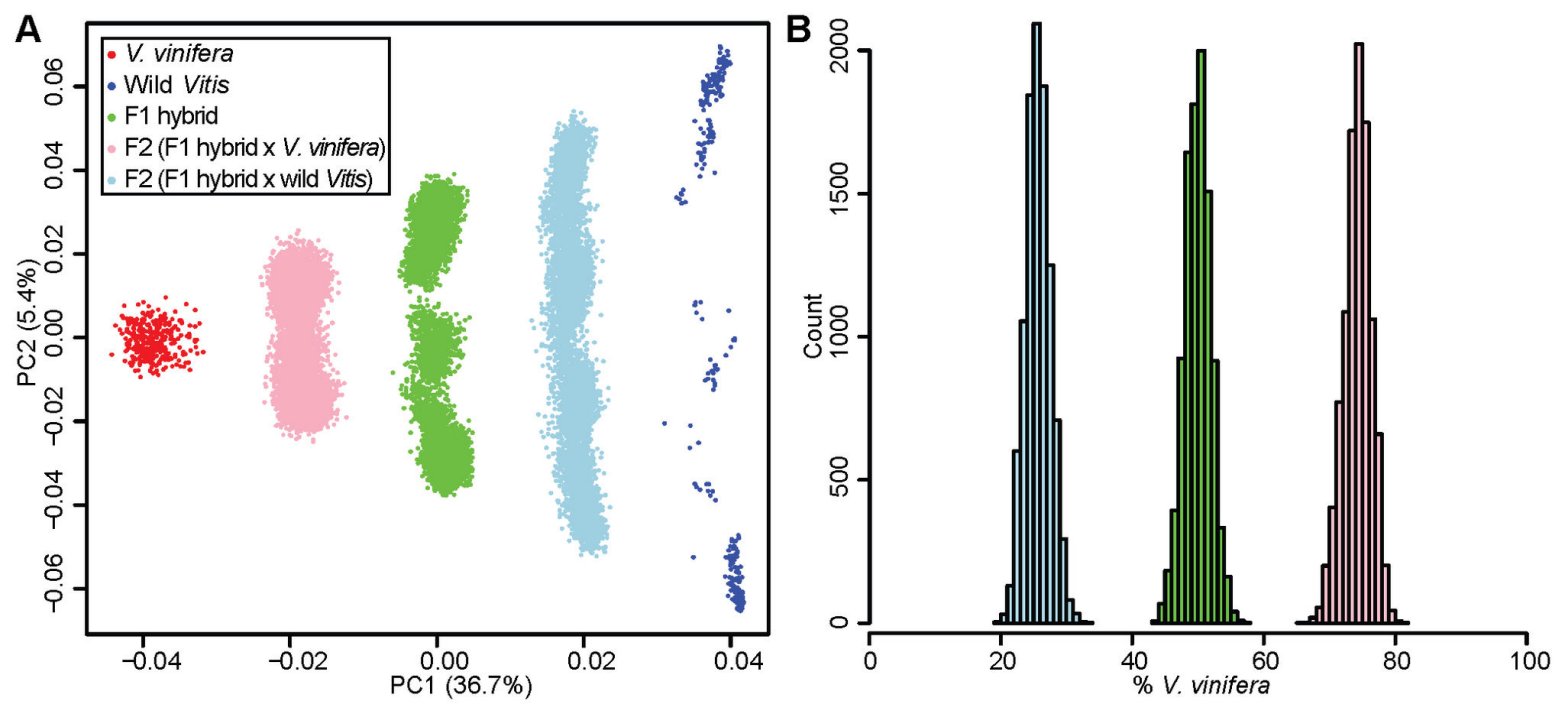
Verification of PCA-based ancestry estimates through simulation. (A) 10,000 F1 hybrids (green) were generated by simulating *V. vinifera* x wild *Vitis* crosses. Using the simulated genotype data, these hybrid samples were then projected onto the PC axes defined by the 333 *V. vinifera* (red) and 333 wild *Vitis* samples (blue) and the proportion of each F1 hybrid’s ancestry derived from each ancestral population was estimated using our PCA-based approach. The same method was applied to F2 populations derived from backcrossing F1 hybrids to *V. vinifera* (pink) and backcrossing F1 hybrids to wild *Vitis* (light blue). (B) The distribution of *V. vinifera* ancestry proportions for the F1 and F2 populations.

 To verify the accuracy of our PCA-based ancestry estimation method, we simulated F1 (*V. vinifera* x wild *Vitis*) and F2 hybrids (F1 simulated hybrids backcrossed to *V. vinifera* or wild *Vitis*) using real genotype calls from the ancestral populations. The PCA plot of the simulated progeny and ancestral populations is shown in Figure 2A. The mean estimated genome-wide proportion of *V. vinifera* in the simulated F1 hybrids was 0.499, 95% CI [0.460, 0.537]. We expect the proportion of *V. vinifera* in these individuals to be 0.5, with the remainder of the genome (0.5) being contributed from the wild *Vitis* population. For offspring of the simulated F1 x *V. vinifera* cross we estimate the mean genome-wide proportion of *V. vinifera* at 0.743, 95% CI [0.699, 0.782], with an expected value of 0.75. For offspring of the simulated F1 x wild *Vitis* cross, we estimate the mean genome-wide proportion of *V. vinifera* at 0.257, 95% CI [0.222, 0.294], with an expected value of 0.25. Distributions of the estimated *V. vinifera* genomic content of the three simulated crosses using PCA-based ancestry estimation are shown in Figure 2B. These results demonstrate that our PCA-based method provides reasonably accurate ancestry estimates for F1 and F2 backcrossed hybrid grape cultivars generated from a highly diverse collection of grape germplasm.

### Grape ancestry estimation

**Figure 3:**
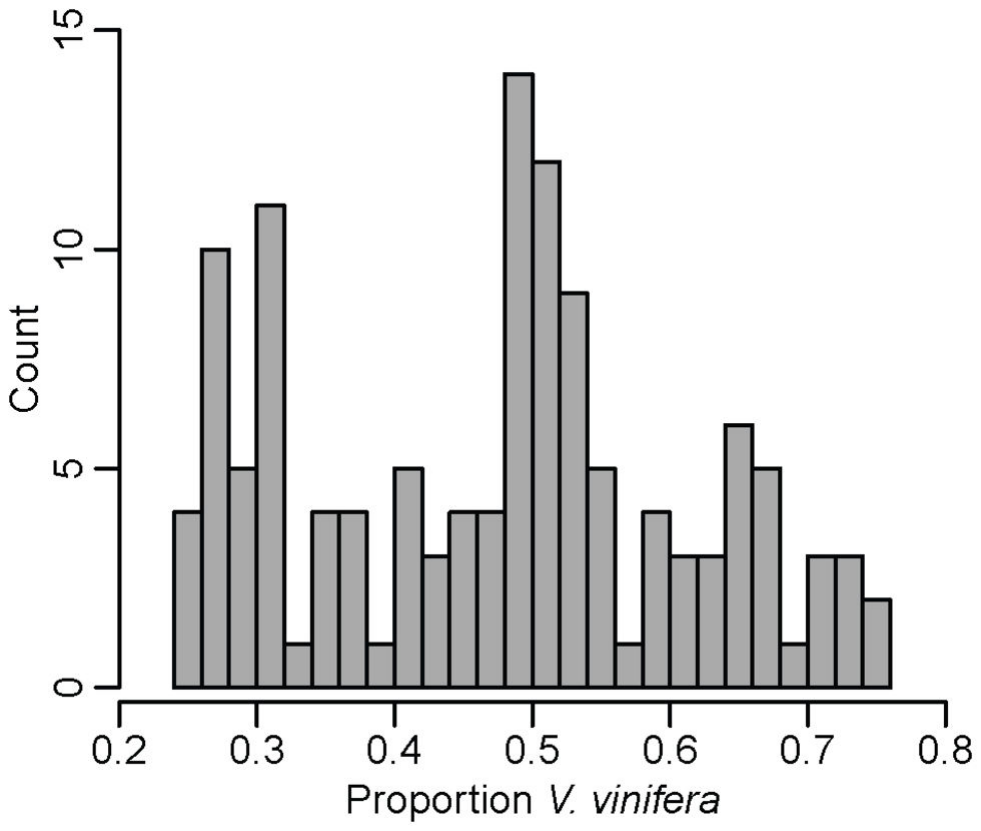
Estimated *V. vinifera* content in grape hybrids. The distribution of *V. vinifera* ancestry proportions in 127 hybrids from the USDA germplasm repository. Estimates are based on the full set of 2959 SNPs. A table of cultivar names, information and proportion *V. vinifera* ancestry is provided in Table S1.

 Genome-wide *V. vinifera* content was estimated for the 127 samples identified as being hybrids with *V. vinifera* and wild *Vitis* ancestry (Figure 3; Table S1). The mean proportion of *V. vinifera* content for these hybrids is 0.474 (Min: 0.2506, Max: 0.7508). The range of observed admixture estimates in hybrid grapes suggests that backcrossing to both wild *Vitis* and *V. vinifera* has occurred in the past century of interspecific grape breeding. The relatively large number of samples with approximately 50% *V. vinifera* contribution to their genome suggests the existence of many first-generation interspecific hybrids in the USDA grape collection. An F1 hybrid included in this analysis, Baco Noir, is a cross between Folle Blanche (*V. vinifera*) and an accession of *Vitis riparia* (North American wild *Vitis* species). Based on its pedigree, we expect ancestry proportions of 50% wild and 50% *V. vinifera*, and our method using the full 2959 SNPs provides estimates of 49% and 51%, respectively. The cultivar Alicante Ganzin is the result of a cross between Alicante Bouschet (*V. vinifera*) and Ganzin No. 4. Ganzin No. 4 is an F1 hybrid between *V. rupestris* (North American wild *Vitis*) and Aramon Noir (*V. vinifera*). This pseudo-backcross pedigree suggests the genome-wide *V. vinifera* content for Alicante Ganzin should be 75%. Our method provides an estimate of 75.1% *V. vinifera* for this sample (Table S1). 

According to the distribution in Figure 3, it appears that, unlike breeders of many other crops, grape breeders have not explicitly aimed to introgress specific genetic loci from wild *Vitis* species by repeatedly backcrossing to the domesticated species, *V. vinifera*. In fact, the large number of cultivars with low % *V. vinifera* ancestry suggests that backcrossing to wild *Vitis* may have been more frequent than backcrossing to *V. vinifera*. However, hybrids with *V. vinifera* content outside a particular range are not included in this analysis due to thresholds established in PC space for hybrid classification (see Methods). If breeders have historically aimed to minimize wild *Vitis* content in hybrid grapes by backcrossing extensively to *V. vinifera*, it is possible that commercially successful hybrid cultivars fall outside our established thresholds and thus may be underrepresented here. In addition, the sample of hybrids from the USDA collection genotyped in the present study may not be representative of interspecific grape breeding in general. Thus, ancestry estimation of a large sample of hybrid grape cultivars from breeding programmes worldwide is currently underway to verify this claim.

When PCA is applied to the genotypes generated from the Vitis9KSNP array, there is a clear distinction between *V. vinifera* and wild North American *Vitis* species along the first principal component (Figure 1A, Figure S1). The genotype data are also sufficient to enable the various wild species to be distinguished from each other. For example, wild *Vitis* samples clearly cluster by species along PC2 (Figure S1). This suggests that our present method could be extended to identify the precise wild *Vitis* species that has contributed to a hybrid’s ancestry: a hybrid’s position on PC2 is likely an indicator of the wild *Vitis* species that has contributed to its ancestry. However, many hybrid grape cultivars have complex pedigrees with genetic contributions from multiple wild *Vitis* species. For example, the hybrid Brianna derives its ancestry from seven different wild *Vitis* species and *V. vinifera* [21]. Extensions of the present method beyond a simple 2-way admixture model and higher density genotype data will be required to generate accurate estimates of the genetic contributions of each individual wild *Vitis* species in complex hybrids. A high-density set of SNPs for this purpose could be generated using a genotyping by sequencing (GBS) approach [22]. 

### Selection of Ancestry Informative Markers (AIMs)

**Figure 4:**
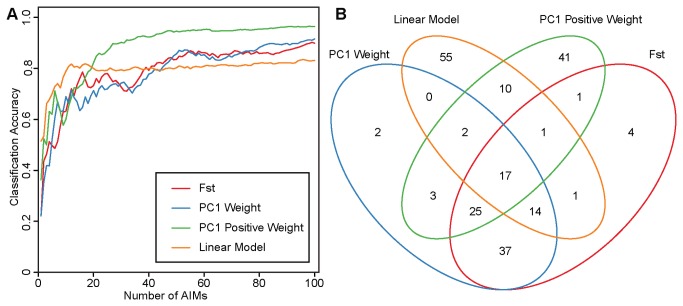
Comparison of measures of informativeness for identifying AIMs. (A) Each measure used to rank AIMs is shown in the legend. For each measure, the proportion of *V. vinifera* ancestry across the 127 hybrids was estimated using 1-100 SNPs ranked according to that measure and the result was compared to the proportion of *V. vinifera* ancestry estimated from the full set of 2959 SNPs. The classification accuracy (Y axis) is the squared Pearson correlation coefficient (R^2^) between the estimate derived from the reduced set of AIMs and the estimate from the full set of SNPs. (B) A Venn diagram showing the overlap of the top 100 AIMs identified using each of the measures.

 To enable ancestry estimation in grapes not included in this study, we investigated several methods for identifying a small number of SNPs, or ancestry informative markers (AIMs), that most effectively capture the ancestry information contained within the full set of 2959 SNPs. In admixed populations, an ideal AIM should have alleles that are fixed between the two ancestral populations and thus have an F_ST_ = 1.0 [23]. In addition, PCA generates weights for each SNP indicating the degree to which a SNP contributes to each PC. SNPs with extreme PC1 weights differentiate *V. vinifera* from wild *Vitis* along PC1 and are thus also good candidate AIMs [11]. We find that Fst values and PC1 weights are highly correlated (R^2^ = 0.979; Figure S3) and that both metrics are useful for the selection of AIMs (Figure 4A). This relationship between Fst and the first principal component has been previously described in [10]. 

We reasoned that the effectiveness of an AIM should not only depend on its frequency difference between the ancestral populations, but also on the extent to which the segregation pattern of its alleles in the hybrid population correlates with the ancestry of the hybrids. Thus, we developed a linear model of informativeness (LM; see Methods) and found that it outperformed both F_st_ and PC1 weights when fewer than 20 SNPs are used, but failed to improve when additional SNPs were added (Figure 4A). 

The PC1 weight of a SNP can be interpreted as a scaled regression coefficient that takes on negative values when PC1 values are negatively correlated with genotypes and positive values when this correlation is positive. The sign of the weight depends on how the genotypes are encoded in the input file. For the present study, genotypes were encoded as follows: 0 = homozygous reference allele; 1 = heterozygous; 2 = homozygous alternative allele. Because the grape reference genome is *V. vinifera* [24], reference alleles are more frequent in the *V. vinifera* samples which occupy the lower values along PC1 (Figure 1). This results in a PC1 weight distribution that is highly skewed towards positive values (Figure S4). Thus, SNPs with extreme negative PC1 weights, where *V. vinifera* are homozygous for the alternative allele and wild *Vitis* species are homozygous for the reference allele, are rare. We reasoned that most of the useful ancestry information would therefore be contained within the positive PC1 weights. We therefore not only tested the ancestry informativeness of markers based on the absolute value of the PC1 weights as is normally done [8,11,25], but also ranked SNPs by their positive PC1 weights only. We found that ignoring the negative weights and only considering the positive weights significantly reduced the number of AIMs required to accurately infer ancestry (Figure 4A). This observation should serve as a cautionary note to future uses of PC1 weights for the purposes of AIM identification.

The physical coordinates of the AIMs identified in the present study can be found in Table S2. Each of the four measures we used to rank SNPs by ancestry informativeness resulted in a different set of AIMs. The overlap in the top 100 AIMs identified by each measure is shown as a Venn diagram in Figure 4B. Within the four sets of 100 markers, 55 and 41 SNPs were unique to LM and PC1 Positive Weight, respectively. The PC1 Weight and F_ST_ panels had 93 SNPs in common. Thus, the selection of AIMs on the basis of PC1 weight and F_ST_ produce highly similar marker panels, however additional informative markers are overlooked if other measures are not taken into consideration. Although each measure is useful in identifying a set of AIMs, there is clearly a need for a method that can conclusively identify the optimal set of AIMs that maximizes ancestry informativeness.

## Conclusions

Over the past century, grape breeders have generated interspecific hybrid grapes by crossing cultivars of the cultivated *V. vinifera* species with numerous wild *Vitis* species. Our PCA-based ancestry estimates of 127 hybrid cultivars indicate that F1 hybrids (*V. vinifera* x wild *Vitis*) are common and that backcrossing to wild *Vitis* was equally or even more frequent than backcrossing to *V. vinifera*. However, estimates from a representative sample of commercial hybrids with known pedigrees are required to verify this claim. Our method provides a framework for enabling marker-assisted breeding of seedling populations based on ancestry estimates, but the application of such background selection in bi-parental populations will require higher marker densities than those provided by the Vitis9KSNP array. 

We identify sets of AIMs and demonstrate that genotypes from only ~50 SNPs are sufficient to accurately estimate the proportion of ancestry a hybrid grape derives from *V. vinifera* and wild *Vitis* species. Not only can the AIMs identified here be employed to curate germplasm collections, but they can also be used for forensic purposes. Regulatory and appellation systems around the world like the AOC (France), DOC (Italy), QmP (Germany) and VQA (Canada) exist to verify and guarantee the authenticity of the origin of their wines. Often, these systems only approve the use of cultivars with 100% *V. vinifera* ancestry, yet the ancestry inferences they employ are often based on questionable morphological analyses, error-prone breeding records or pure conjecture. Although it is widely recognized by the scientific community that the restriction of cultivar use by these organizations poses a serious threat to the future of the wine and grape industry [4], the set of AIMs and the method presented here provide a robust forensic tool that can be used to definitively verify the ancestry criteria these regulatory agencies attempt to apply. 

## Supporting Information

Figure S1 **PCA of 1599 samples from USDA grape germplasm collection.** (A) PC axis 1 (PC1) and PC2 were calculated using 2959 SNPs from 333 *V. vinifera* and 333 wild Vitis samples. The proportion of the variance explained by each PC is shown in parentheses along each axis. Subsequently, 1599 samples, including various *Vitis* species and hybrids, were projected onto these axes. This is the same plot as Figure 1 in the main manuscript, but each sample is labeled with the species identifier associated with that sample. Species identifiers were obtained from the Germplasm Resources Information Network (GRIN) database managed by the USDA. It is evident that many samples are mislabeled. For example, some samples labeled as *V. vinifera* clearly cluster far to the right of PC1 with the wild species. In cases where there was an obvious error and it interfered with downstream analyses, the samples were removed from analysis (N = 60). Eurasian wild Vitis samples and hybrids with known ancestry from Eurasian wild species were removed from the analysis. See Materials and Methods on how we defined “hybrid” for the present study.
(PDF)

Figure S2 **A comparison of ancestry estimates derived from our PCA-based method and the model-based method STRUCTURE.** The proportion *V. vinifera* ancestry estimated using the PCA-based method and the programme STRUCTURE are shown on the X and Y axes, respectively, for the 127 hybrid samples analysed in the present study.
(PDF)

Figure S3 **(**A**) F_ST_ and the absolute value of PC1 weights are highly correlated.** (B) The classification accuracy of AIMs ranked by Fst and PC1 absolute weight are highly similar.
(PDF)

Figure S4 **The distribution of PC1 weights from running SMARTPCA on 333 *V. vinifera* and 333 wild *Vitis* samples.** The distribution is skewed towards positive values.
(PDF)

Table S1 **A list of the 127 accessions from the USDA grape germplasm collection considered “hybrid” in the current study based on their positions along PC1 and associated cultivar name for these accessions from the USDA Germplasm Resources Information Network (GRIN) database.**
(XLS)

Table S2 **A list of the AIMs identified in the present study.** The top 100 AIMs are listed for each of the four measures used in the present study. The AIMs are ranked according to the measure listed at the top of each column. The name of each SNP contains the physical coordinates of the SNP according to the 8x Pinot Noir reference genome, where the chromosome name is separated by the physical position by a colon. Chromosome numbers outside of the range of 1-19 refer to the unanchored contigs found in the 8x Pinot Noir reference genome.
(XLS)
