# Supplementary figures and images for: Genomics Assisted Ancestry Deconvolution in Grape
Source: PLoS One. 2013 Nov 11;8(11):e80791. doi: 10.1371/journal.pone.0080791 (PMC3823699; doi:10.1371/journal.pone.0080791)

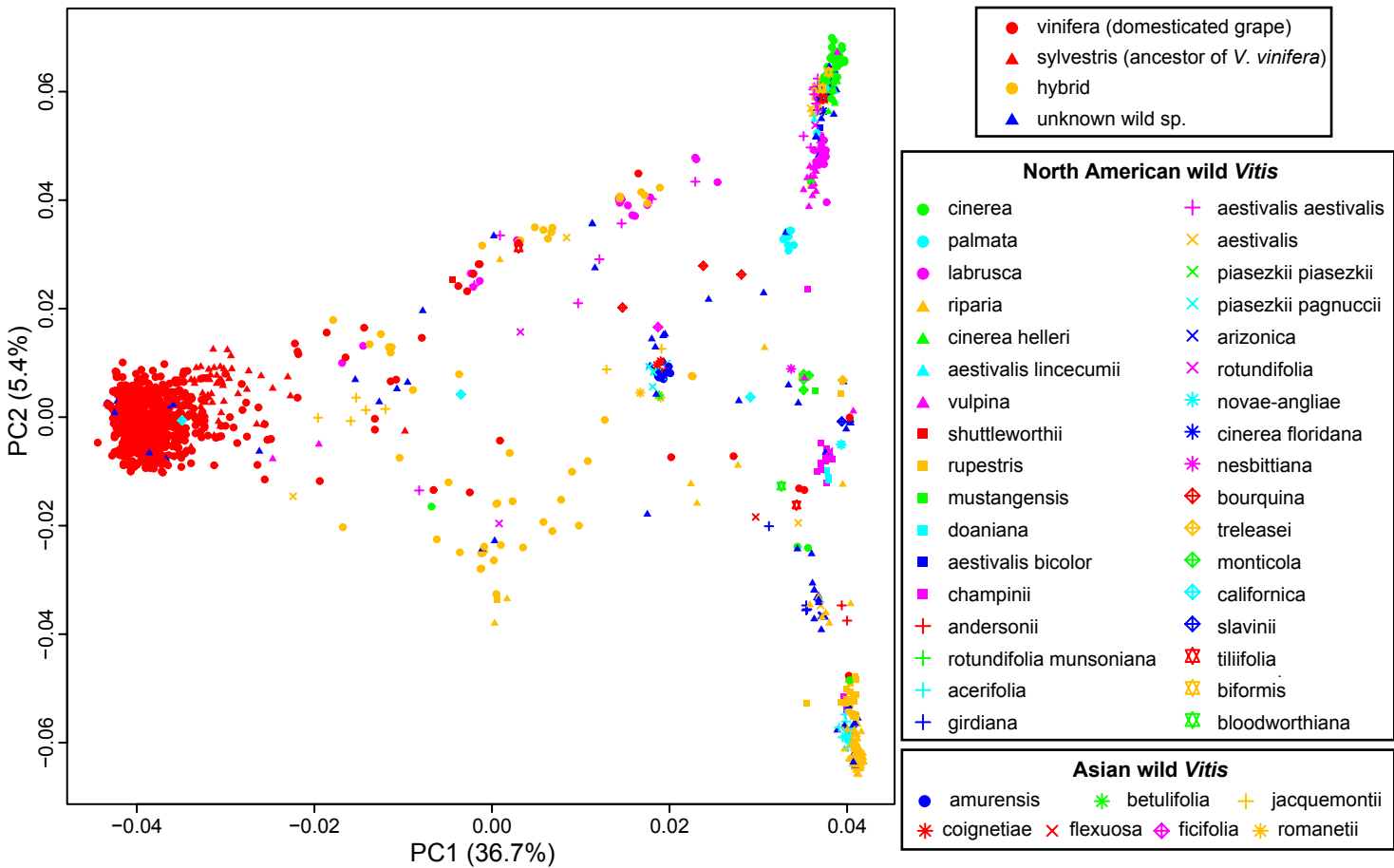

Supplement: Figure S1 — PCA of 1599 samples from USDA grape germplasm collection. (A) PC axis 1 (PC1) and PC2 were calculated using 2959 SNPs from 333 V. vinifera and 333 wild Vitis samples. The proportion of the variance explained by each PC is shown in parentheses along each axis. Subsequently, 1599 samples, including various Vitis species and hybrids, were projected onto these axes. This is the same plot as Figure 1 in the main manuscript, but each sample is labeled with the species identifier associated with that sample. Species identifiers were obtained from the Germplasm Resources Information Network (GRIN) database managed by the USDA. It is evident that many samples are mislabeled. For example, some samples labeled as V. vinifera clearly cluster far to the right of PC1 with the wild species. In cases where there was an obvious error and it interfered with downstream analyses, the samples were removed from analysis (N = 60). Eurasian wild Vitis samples and hybrids with known ancestry from Eurasian wild species were removed from the analysis. See Materials and Methods on how we defined “hybrid” for the present study. (PDF) [file pone.0080791.s001.pdf]

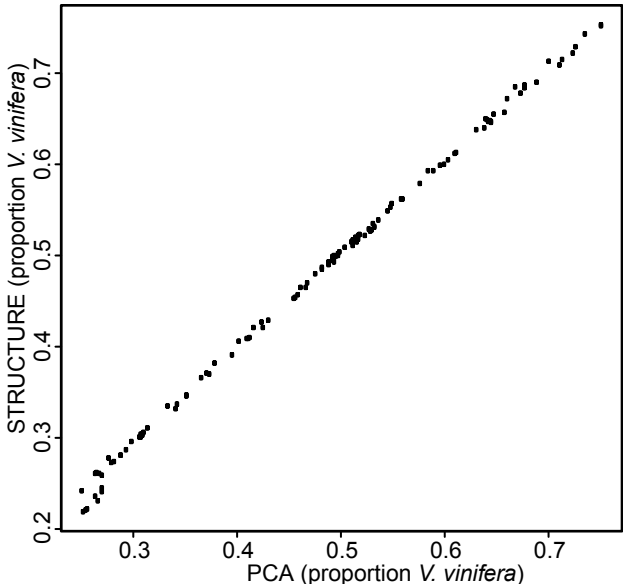

Supplement: Figure S2 — A comparison of ancestry estimates derived from our PCA-based method and the model-based method STRUCTURE. The proportion V. vinifera ancestry estimated using the PCA-based method and the programme STRUCTURE are shown on the X and Y axes, respectively, for the 127 hybrid samples analysed in the present study. (PDF) [file pone.0080791.s002.pdf]

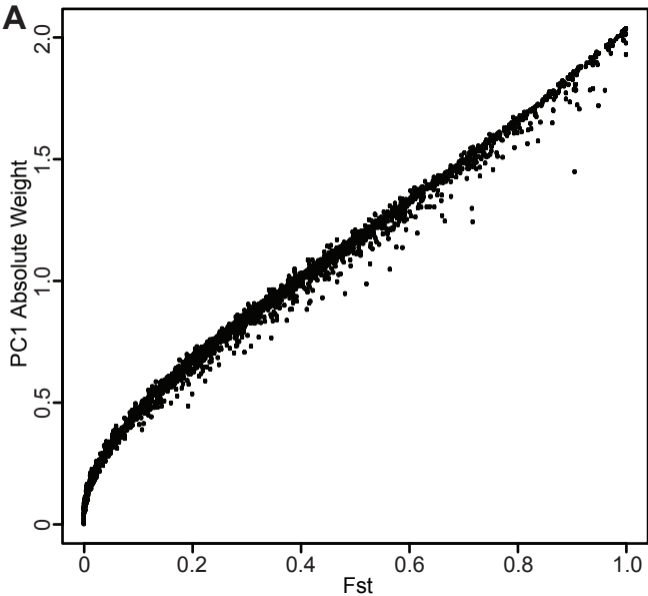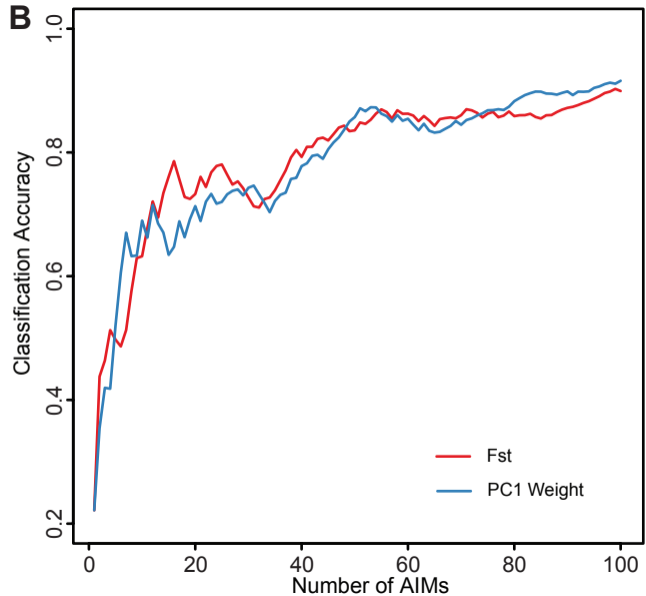

Supplement: Figure S3 — (A) FST and the absolute value of PC1 weights are highly correlated. (B) The classification accuracy of AIMs ranked by Fst and PC1 absolute weight are highly similar. (PDF) [file pone.0080791.s003.pdf]

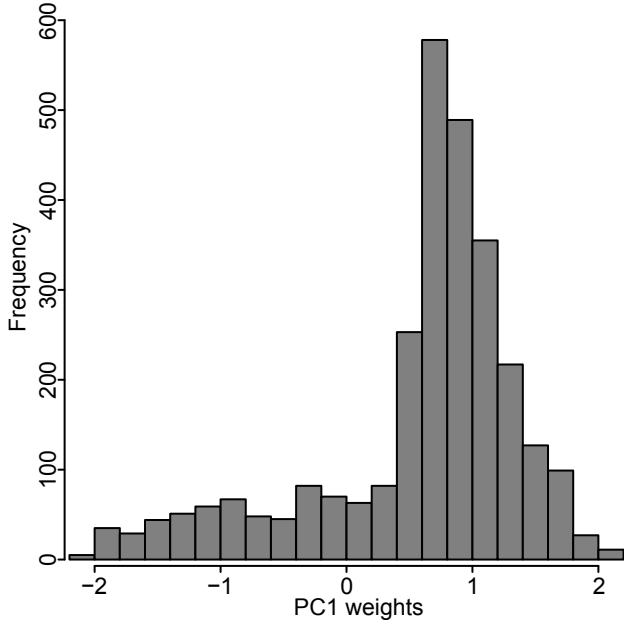

Supplement: Figure S4 — The distribution of PC1 weights from running SMARTPCA on 333 V. vinifera and 333 wild Vitis samples. The distribution is skewed towards positive values. (PDF) [file pone.0080791.s004.pdf]
